# A long noncoding RNA AB073614 promotes tumorigenesis and predicts poor prognosis in ovarian cancer

**DOI:** 10.18632/oncotarget.4541

**Published:** 2015-07-30

**Authors:** Zhongping Cheng, Jing Guo, Li Chen, Ning Luo, Weihong Yang, Xiaoyan Qu

**Affiliations:** ^1^ Department of Obstetrics and Gynecology, Yangpu Hospital, Tongji University School of Medicine, Shanghai, PR China; ^2^ Institute of Gynecological Minimally Invasive Medicine, Tongji University School of Medicine, Shanghai, PR China

**Keywords:** lincRNA, AB073614, ovarian cancer, RNAi

## Abstract

Long noncoding RNA (lncRNA) profiles in ovarian cancer (OC) remain largely unknown. In the present study, we screened AB073614 as a new candidate lncRNA which promotes development of OC, in two independent datasets (GSE18521 and GSE38666) from the Gene Expression Omnibus (GEO). The level of AB073614 was then detected in 75 paired OC tissues and adjacent normal tissues by qRT-PCR. Results showed that AB073614 expression was significantly up-regulated in 85.3% (64/75) cancerous tissues compared with normal counterparts (*P* < 0.01). Further, the 5-year overall survival (OS) in OC patients with high expression of AB073614 was inferior to that with low expression (17.2 months *vs* 30.0 months, *P* = 0.0025). To investigate the functional role of AB073614, AB073614 siRNA was transfected into OC cell lines. Knockdown of AB073614 expression significantly inhibited cell proliferation and invasion, resulted in cell arrest in G_1_ phase of cell cycle and a dramatic increase of apoptosis, both in HO-8910 and OVCAR3 cells. *In vivo* experiment also revealed that knockdown AB073614 inhibited OVCAR3 cells proliferation. Finally, western blot assays indicated that lncRNA AB073614 may exert its function by targeting ERK1/2 and AKT-mediated signaling pathway. In conclusion, our study suggests that lncRNA AB073614 acts as a functional oncogene in OC development.

## INTRODUCTION

Ovarian cancer (OC) is the most lethal gynecological cancer and a common cause of cancer-related deaths in women worldwide [1–3]. Despite advances in surgery and chemotherapy, the overall survival of OC patients remains unsatisfactory, with a five-year survival rate of only 30% [4]. Patients with this malignancy have an extremely poor prognosis due to late clinical presentation, subtle symptomatology, and rapid disease progression. In order to develop better preventive and diagnostic approaches, as well as more effective treatment modalities, a deep understanding is required of the molecular mechanisms implicated in the complex process of ovarian carcinogenesis.

Long non-coding RNAs (lncRNAs, >200 nt in length), initially thought to represent spurious transcriptional noise, have acquired extensive attention recently as new regulators in many biological processes from nuclear organization to epigenetic modification of post-transcriptional regulation and RNA splicing [5]. Emerging evidences indicate that lncRNAs may play complex and extensive roles in promoting the development and progression of cancer [6, 7].

Although the dysregulation of lncRNAs have been shown in OC, such as HOTAIR, HOST2 [8, 9], lncRNA profiles in this malignancy remain largely unknown. Previous study demonstrated that lncRNA profiling could be achieved by mining previously published gene expression microarray data because a large group of lncRNA-specific probes were fortuitously represented on the commonly used microarray platforms [10, 11]. In the present study, GATExplorer (Genomic and Transcriptomic Explorer) [12] was used to process microarrays on a local computer for gene expressions of lncRNAs profiling (GSE18521 and GSE38666) on two independent datasets from the Gene Expression Omnibus (GEO). By applying comprehensive analysis on LncRNA expression profiles, we identified AB073614 as a new candidate lncRNA which promotes development of OC. Further, its biological role and clinical significance were evaluated.

## RESULTS

### Screen of OC specific LncRNA

**Figure 1:**
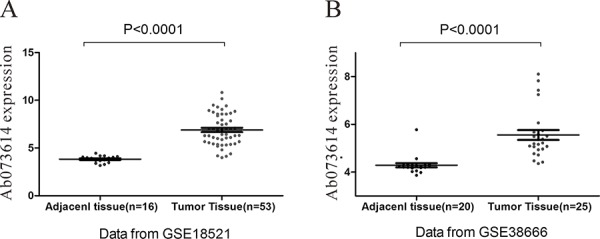
Screen of OC specific LncRNA in GEO database LncRNA AB073614 was consistently over-expressed in OC tissue compared to the normal tissue in both of GSE18521 **(A)** and GSE38666 **(B).**

Arraytools (http://linus.nci.nih.gov/BRB-ArrayTools.html) was used to search the differential expressed lncRNA between OC tissue and normal tissue. Here, we found lncRNA AB073614 was consistently over-expressed in OC tissue compared to the normal tissue in both of GSE18521 and GSE38666 database (Figure 1) (*P* < 0.001).

### Expression of AB073614 is up-regulated in OC tissues

**Figure 2:**
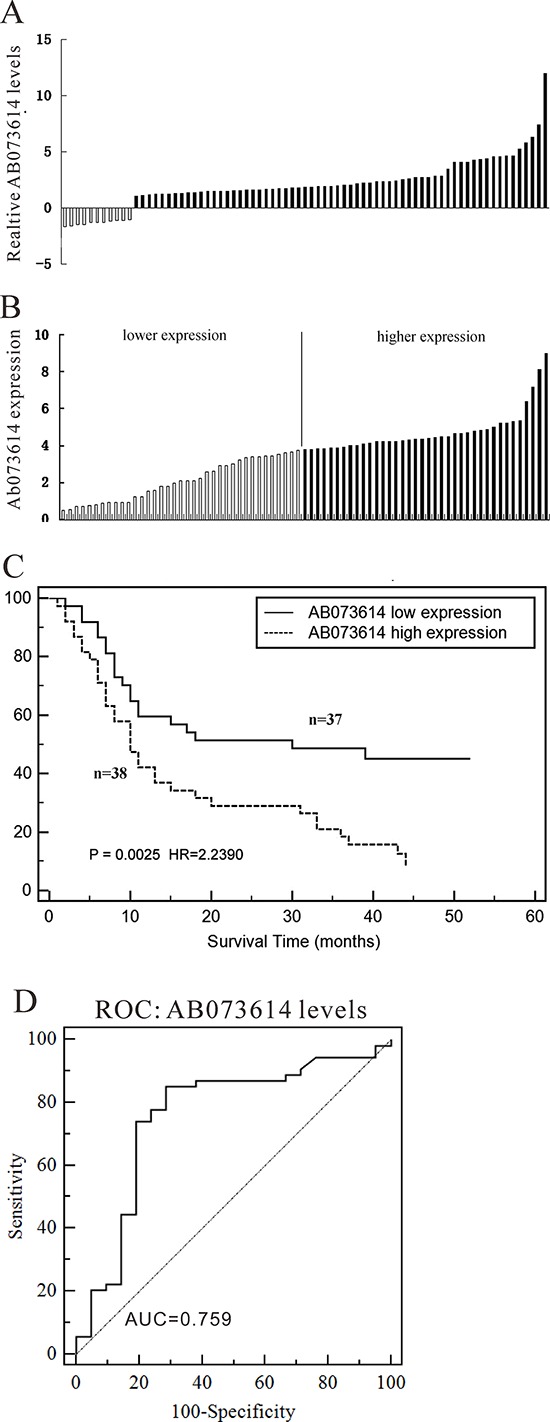
LncRNA AB073614 expression in human ovarian cancer tissues **A.** Relative expression of AB073614 in OC tissues (*n* = 75) compared with corresponding non-tumor tissues (*n* = 75). AB073614 expression was examined by qPCR and normalized to GAPDH expression. Results are presented as the fold-change in tumor tissues relative to normal tissues. **B.** LncRNA AB073614 expression was classified into two groups, according the expression level in OC tissues. **C.** The correlation between AB073614 expression and prognosis. 5 year overall survival was analyzed by Kaplan-Meier survival curve. **D.** The receiver operating characteristic (ROC) curve for prognosis prediction of patients using AB073614 level. The area under curve (AUC) was shown in the plots.

The level of AB073614 was detected in 75 paired OC tissues and adjacent normal tissues by qRT-PCR, and normalized to GAPDH. AB073614 expression was significantly up-regulated in 85.3% (64/75) cancerous tissues compared with normal counterparts (*P* < 0.01) (Figure 2A). Further, according to the relative AB073614 expression in tumor tissues, the 75 OC patients were classified into two groups: relative high group (*n* = 38) and relative low group (*n* = 37) (Figure 2B). Kaplan-Meier analysis and log-rank test were used to evaluate the correlation of AB073614 expression and prognosis, as shown in Figure 2C, the 5-year OS in OC patients with high expression of AB073614 was inferior to that with low expression (mean 17.2 months (95% CI: 12.353–22.005) *vs* 30.0 months (95% CI: 23.193–36.823), *P* = 0.0025). Furthermore, the receiver operating characteristic (ROC) curve analysis revealed that AB073614 level was useful to predict patient survival of OC (Figure 2D, area under curve [AUC]: 0.759, 95% CI: 0.647–0.851).

### Knockdown AB073614 inhibits OC cells proliferation and invasion *in vitro*

**Figure 3:**
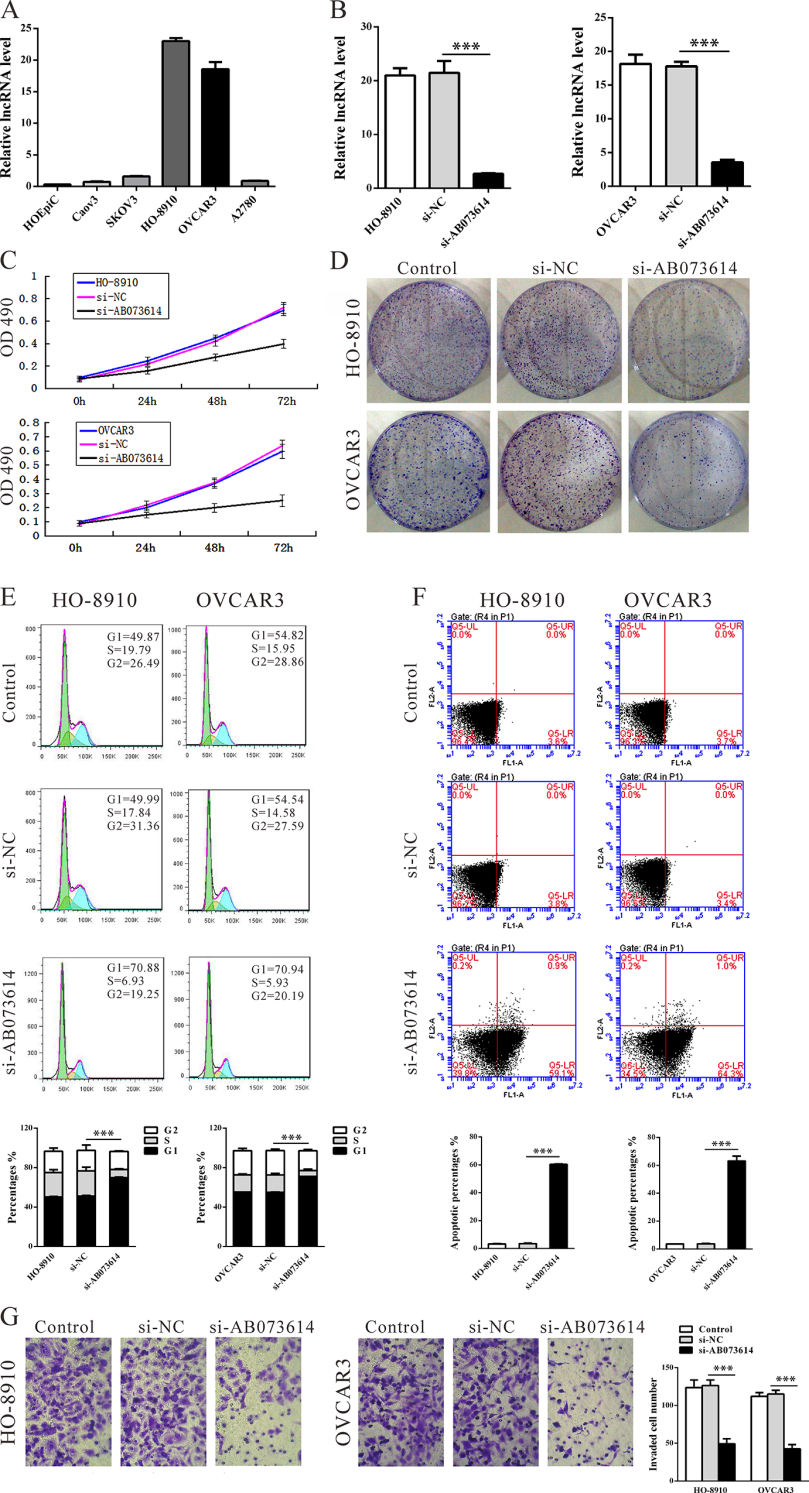
Knockdown AB073614 inhibits OC cells proliferation and invasion *in vitro* **A.** Expression of lncRNA AB073614 in human ovarian epithelial cell line (HOEpiC) and five OC cell lines as determined by qRT-PCR. **B.** Knockdown efficiency was determined by qRT-PCR in HO-8910 and OVCAR3 cells. Knockdown AB073614 in HO-8910 and OVCAR3 cells significantly reduced their proliferative capacities, as determined by cell number counting assay **C.** and colony formation assay **D.** Knockdown AB073614 in HO-8910 and OVCAR3 cells resulted in cell arrest in G1 phase of cell cycle **E.** and a dramatic increase of apoptosis **F.** Invasion and metastasis capacities determined by Transwell assays **G.**

To investigate the functional role of AB073614 in OC cells, firstly, qRT-PCR was performed to detect the expression of AB073614 in diverse OC cell lines. As shown in Figure 3A, AB073614 expression level was increased significantly in 5 cancer cell lines, compared with a human ovarian epithelial cell line (HOEpiC). Then AB073614 siRNA was transfected in to HO-8910 and OVCAR3 cell lines, which have the highest levels of AB073614. qPCR assays revealed that AB073614 expression was significantly reduced in two cell lines (Figure 3B). MTT and colony-formation assay showed that knockdown of AB073614 expression significantly inhibited cell proliferation both in HO-8910 and OVCAR3 cell lines compared with the control cells (Figure 3C, 3D).

Next, flow cytometric analysis revealed that the knockdown AB073614 resulted in cell arrest in G_1_ phase of cell cycle (Figure 3E) and a dramatic increase of apoptosis (Figure 3F) both in HO-8910 and OVCAR3 cell lines. Finally, transwell assays showed the number of HO-8910 or OVCAR3 cells in lower section were significantly reduced in the AB073614 knockdown groups compared with the control groups (Figure 3G), which indicated that the expression of lncRNA AB073614 promote cell invasion and metastasis.

### Knockdown AB073614 inhibits OC cells proliferation *in vivo*

**Figure 4:**
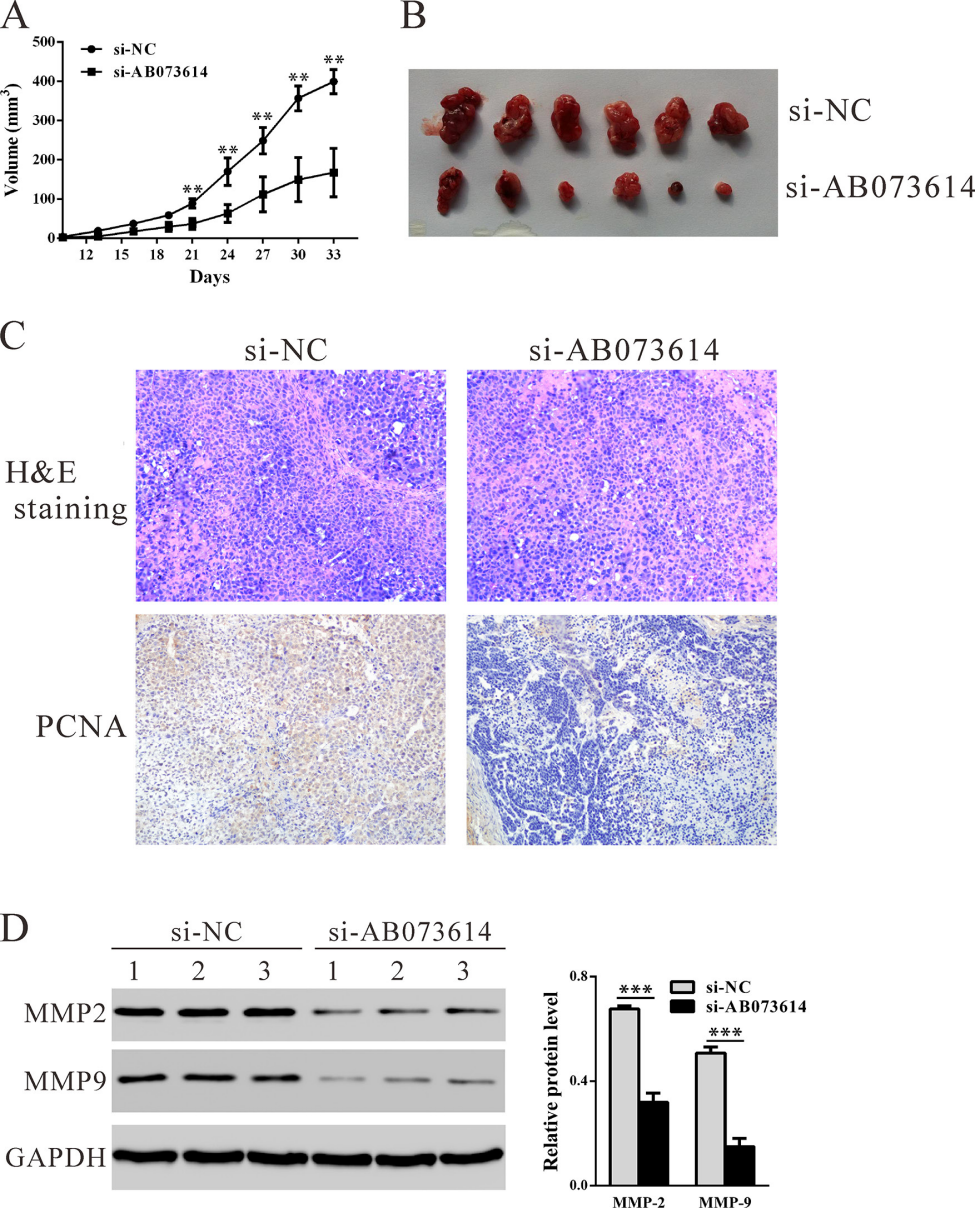
Knockdown AB073614 inhibits OC cells proliferation *in vivo* OVCAR3 cells transfected with Si-AB073614 or Si-NC were subcutaneously inoculated into nude mice (6 per group). **A.** The tumor size was monitored every three days. **B.** Mice were sacrificed and the tumors were isolated after 33 days. **C.** Transplanted tumors with H&E staining and PCNA immunostaining. **D.** The expression of MMP-2 and MMP-9 in xenograft from the nude mice was determined by western blot.

To confirm the effect of lncRNA AB073614 *in vivo*, OVCAR3 cells transfected with Si-AB073614 or Si-NC were subcutaneously inoculated into nude mice. As shown in Figure 4A, tumors derived from knockdown AB073614 cells grew more slowly in comparison with the control ones. All mice were sacrificed 33 days after the transplantation, the average size of tumors derived from knockdown AB073614 cells was significantly reduced 58.1% (*P* = 0.007, Figure 4B). Further, immunostaining revealed that the proliferating cell nuclear antigen (PCNA)-positive cells were significantly decreased in tumors formed from knockdown AB073614 cells compared with that in NC-ones (Figure 4C). Western blot analysis revealed that invasion related proteins, MMP2 and MMP9, were also significantly decreased in tumors formed from knockdown AB073614 cells (Figure 4D).

### Mechanisms of lncRNA AB073614 exerts its function

**Figure 5:**
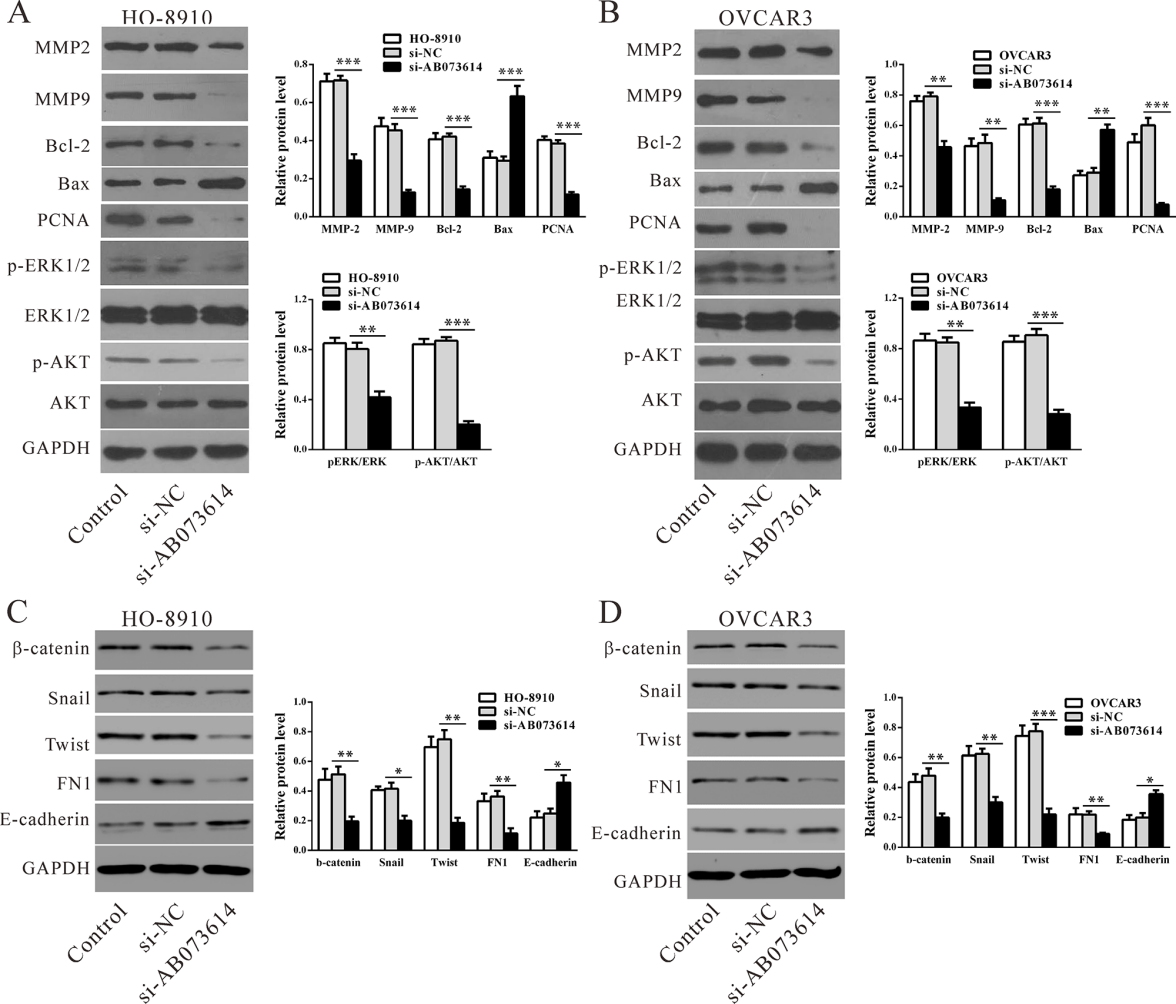
Mechanisms of LncRNA AB073614 exerts its function Signal pathway and key moderators in tumor progression were determined by western blotting in HO-8910 **A, C.** and OVCAR3 cells **B, D.** Left panel, representative results of western blot; right panal, protein levels relative to GAPDH. Data were presented as the mean value from three independent experiments ± S.D. **P < 0.01, **P < 0.001.

To analyze the expression of genes downstream of lncRNA AB073614, western blot assays were performed (Figure 5). Consistent with functional characterization *in vitro*, knockdown AB073614 caused cell growth (PCNA), metastasis (MMP-2, 9), epithelial-mesenchymal transition (EMT) (β-catenin, Twist, Snail and FN1), anti-apoptosis (Bcl-2) related factors downregulated significantly both in HO-8910 and OVCAR3 cell lines compared with the control cells, while apoptosis factor (Bax) and the main factor of EMT (E-cadherin) significantly upregulated. Moreover, p-ERK1/2 and p-AKT protein expression were significantly reduced in the knockdown AB073614 group compared with the controls, both in HO-8910 and OVCAR3 cell lines, while ERK1/2 and AKT protein expression were not changed, which indicated that lncRNA AB073614 may exert its function by inhibition ERK1/2 and AKT-mediated signaling pathway.

## DISCUSSION

The conventional view of gene regulation in biology has centered on protein-coding genes until the discovery of thousands of lncRNAs. Numerous reports of dysregulated lncRNA expression across numerous cancer types suggest that abnormal lncRNA expression may be a major contributor to tumorigenesis [15–17]. The aberrant expressions of specific lncRNAs in cancer could mark the spectrum of disease progression and these lncRNAs may serve as independent biomarkers for diagnosis and prognosis [18, 19].

Presently, a large group of lncRNA-specific probes were fortuitously represented on the commonly used microarray platform (Affymetrix HG-U133 plus 2.0), so we initially mined previously published gene expression microarray data from GEO database, and conducted lncRNA profiling on large cohorts of OC patients. In this study, we identified AB073614 was most significantly upregulated one in cancerous tissues. To further confirm the reliability of the new signature we conducted a series of experiments to investigate the roles of lncRNA AB073614 in OC development.

LncRNA AB073614 is a new lncRNA transcript which first be considered as a Homo sapiens primary hepatoblastoma cDNA (clone: HMFN1050) [20]. It remains unclear whether lncRNA AB073614 has important biological functions. In the present study, we confirmed that lncRNA AB073614 is extremely upregulated in 75 OC tissues and 5 cancer cell lines, compared with normal tissues and ovarian epithelial cell line. Importantly, the OS of patients with higher AB073614 expression levels was shorter than that with lower expression. These findings indicate that lncRNA AB073614 plays a direct role in the modulation of OC progression and may be considered as a novel prognostic marker for OC.

To further investigate the functions of lncRNA AB073614 in OC, we explored the effects of loss of function on various aspects of OC cell biology. Firstly, two OC cell lines (HO-8910 and OVCAR3) with higher AB073614 expression were chosen. RNAi-mediated suppression of lncRNA AB073614 in both cell lines significantly inhibited cells proliferation, migration and invasion, and induced cell cycle G_1_ phase arrest and apoptosis. Growth inhibition effects have also been confirmed in nude mice experiment that tumors derived from knockdown AB073614 cells grew more slowly. While, overexpression of AB073614 in lower expression Caov3 cells accelerated cell cycle transition, and suppressed cell apoptosis and cell invasion (Figure S1). Thus, lncRNA AB073614 plays an oncogenic role in OC and represents a potential target for OC treatment.

To date, the molecular mechanisms by which lncRNAs promote tumor development and metastasis are not fully understood. Possible RNA-targeting schemes include sequence-specific recognition (RNA-RNA), RNA-DNA hybrids, structure-mediated interactions and protein-mediated interactions [21–24]. In some cases, many lncRNAs are processed to yield small RNAs or, conversely, modulate how other RNAs are processed [25]. Many reports have suggested that the oncogenic activity of some lncRNAs might be attributed to miRNAs. Others demonstrated that lncRNAs promote cancer progression by regulating the expression of protein-coding genes. For instance, MALAT-1 regulates the expression of a series of apoptosis-related and EMT-associated genes [26]. These findings prompted us to determine whether lncRNA AB073614 promotes OC growth and metastasis by regulating the expression of genes encoding proteins. As expected, knockdown AB073614 caused cell growth (PCNA), invasion (MMP-2, 9), EMT (β-catenin, Twist, Snail and FN1), and anti-apoptosis (Bcl-2) related factors downregulated significantly both in HO-8910 and OVCAR3 cell lines, while apoptosis factor (Bax) and the main factor of EMT (E-cadherin) significantly upregulated, implying that these genes participate in AB073614-induced OC progression. Further, downregulation of p-ERK1/2 and p-AKT in AB073614-silenced cells and upregulation of p-ERK1/2 and p-AKT in AB073614-overexpressed cells (Figure S2) indicated that lncRNA AB073614 may exert its function by targeting ERK1/2 and AKT-mediated signaling pathway. Additional investigations are required to understand the exact molecular mechanisms by which lncRNA AB073614 regulates these genes and signaling pathways.

In conclusion, the present study, for the first time, suggests that lncRNA AB073614 acts as a functional oncogene in OC cell line, and the up-regulation of lncRNA AB073614 expression is closely associated with OC development. Thus, these results indicate that lncRNA AB073614 may become a novel promising candidate for the prognosis and therapy for OC.

## MATERIALS AND METHODS

### Microarray data of OC

Affymetrix Human Genome U133plus2 Array for the OC and normal tissue were downloaded from the GEO: GSE18521 and GSE38666. GSE18521 consisted of 53 papillary serous ovarian adenocarcinoma and 16 normal tissue and GSE38666 consisted of 25 ovarian tumor tissue and 20 normal tissue. The raw CEL files were downloaded from GEO database and background were adjusted using Robust Mult-ichip Average. GATExplorer was used to process microarrays on a local computer for gene expressions of lncRNAs, which has been used by Hu et.al [13] and Chen et.al [14]. This GATExplorer provides a series of Rpackages, designed to be used with BioConductor tools, which allow to apply in a simple way the probe mapping data included in GATExplorer. A type of files called lncRNA Mapper were also obtained from GATExplorer, which include the probes that do not map to any codingregion but that were mapped to a database for non-codingRNA of human and mouse derived from RNAdb. A customized R scripts was used to perform a microarray expression calculation according to the re-mapping data (file ncrnamapperhgu133acdf_3.0). Each lncRNA should include at least a minimum of 3 probesmapping in the corresponding lncRNAs entity.

### Tissue samples

A total of 75 patients enrolled in this study underwent resection of the primary OC at Yangpu Hospital, Tongji University (Shanghai, China). The study was approved by Research Ethics Committee of Tongji University and written informed consent was obtained from all patients. Tumor samples and according normal tissues were immediately frozen in liquid nitrogen and kept at −80°C until used. Overall survival (OS) was defined as the interval between the dates of surgery and death.

### Cell lines

A human ovarian epithelial cell line (HOEpiC) and five human ovarian cancer cell lines (A2780, Caov3, HO-8910, OVCAR3, and SKOV3) were obtained from Chinese Type Culture Collection, Chinese Academy of Sciences, and were cultured in RPMI 1640 medium (Invitrogen), supplemented with 10% fetal bovine serum (Hyclone), 100 U/ml penicillin sodium, and 100 mg/ml streptomycin sulfate at 37°Cin a humidified air atmosphere containing 5% CO2. Cells were used when they were in the logarithmic growth phase.

### RNA isolation and quantitative RT-PCR

Total RNA was extracted from tissues and cell lines using Trizol reagent (Invitrogen) according to the manufacturer's instructions. The expression levels of AB073614 were determined by quantitative RT-PCR using the SYBR^®^Green (TaKaRa) on ABI StepOne PCR instrument, with GAPDH as an internal control. The primers are as follows: LncRNA AB073614, 5′-ATTTCTGCTCCTGGGTCTTAC-3′ and 5′-AGTGGCTTGTCTGTTAGAGTC-3′; GAPDH (NM_001256799.1), 5′-CACCCACTCCTCCACCTTTG-3′ and 5′-CCACCACCCTGTTGCTGTAG-3′. Comparative Ct method was used for quantification of the transcripts. The fold-change for target genes normalized by internal control was determined by the formula 2^−ΔΔCT^.

### RNA interference

siRNA targeting human AB073614 and one negative control siRNA were designed and synthesized by Genepharma (Shanghai, China). Transfection of each siRNA (50 nM) was carried out using Lipofectamine2000 (Invitrogen) according to the manufacturer's recommendations.

### Cell proliferation assay

To determine cell growth, 2 × 10^3^ cells were seeded in 96-well plate and transfected with siRNA. The proliferation of cell was assessed after siRNA transient transfection for 24, 48 and 72 h. MTT (5 g/L, 20 μL/well) was added to each well and incubated at 37°C for 4 h. DMSO was then added (150 μL/well) to each well to dissolve any crystals and the plates were agitated for 10 min. Absorbance values at 492 nm were detected by the microplate reader (BioTek, VT, United States).

For colony formation assay, five hundred cells per well were seeded in six-well plates and transfected, with siRNA. Two weeks later, colonies were fixed with methanol and stained by 0.1% crystal violet. The colonies with the diameter greater than 1 mm were counted.

### Flow cytometric analysis

Cells were harvested directly or 48 h after siRNA transient transfection and washed with ice-cold phosphate-buffered saline (PBS). The PI/RNase staining kits (Multisciences, Hangzhou, China) and annexin V-fluorescein isothiocyanate (FITC) apoptosis detection kits (KeyGEN Biotech, Nanjing, China) were used to detect cell cycle and apoptosis in a FACScan instrument (Becton Dickinson,, Mountain View, CA, USA), respectively.

### *In vitro* cell invasion assays

Cells were transfected with 50 nM Si-AB073614 or Si-NC. Twenty-four hours post-infection, the infected cells were harvested and plated (1 × 10^5^) in the top chamber of Transwell assay inserts (Millipore, Billerica, MA) with a Matrigel-coated membrane containing 8-μm pores in 200 mL of serum-free RPMI 1640 medium. The inserts were then placed into the bottom chamber of a 24-well plate containing RPMI 1640 with 10% FBS as a chemo-attractant. After 24 h, the top layer of the insert was scrubbed with a sterile cotton swab to remove any remaining cells. The invading cells on the bottom surface were stained with 0.1% crystal violet, examined, counted, and imaged using digital microscopy.

### *In vivo* experiments

The animal study protocol was approved by the Animal Experimentation Ethics Committee of the Tongji University affiliated Yangpu Hospital. Female athymic Balb/c nude mice (aged five weeks, weighing 20–22 g) were provided by Slac Laboratory Animal Co. Ltd. (Shanghai, China). The mice were housed in a pathogen-free animal facility and randomly assigned to the control or experimental group (six mice per group). OVCAR3 cells transfected with Si-NC or Si-AB073614 were harvested and injected intraperitoneally into each mouse (2 × 10^6^/0.2 ml). Tumor volume was estimated every three days by the formula: 0.5 × length × width^2^. All mice were sacrificed after 33 days. Tumor tissues were excised, paraffin-embedded, formalin-fixed, and performed H&E staining and PCNA Immunostaining.

### Western blot assays

Western blot assays were performed using the following primary antibodies: anti-human MMP2 (Abcam, Cambridge, MA, USA; 1:500), Twist (Abcam; 1:500), fibronectin1 (FN1, Abcam; 1:500), MMP9 (cell signaling, Danvers, MA, USA; 1:500), Bcl2 (cell signaling; 1:500), Bax (cell signaling; 1:500), PCNA (cell signaling; 1:500), E-cadherin (Santa Cruz Biotech., Santa Cruz, CA, USA; 1:500), β-catenin (cell signaling; 1:1000), ERK1/2 (cell signaling; 1:1000), p-ERK1/2 (cell signaling; 1:1000), AKT (cell signaling; 1:1000), p-AKT(cell signaling; 1:1000) and GAPDH (Millipore; 1:1,000). Briefly, stimulated cells were lysed with RIPA buffer (50 mM Tris-HCl [pH 7.5], 150 mM NaCl, 1% Triton X-100, 0.5% Na-deoxycholate) containing protease inhibitors; 20–30 μg samples of the lysates were separated on 8%–12% SDS PAGE gels and transferred to PVDF membranes. The membranes were incubated with primary antibodies overnight at 4°C. The primary antibody incubation was followed by incubation with an HRP-conjugated secondary antibody. Finally, the bound antibodies were detected using an ECL substrate.

### Statistical Analysis

The SPSS 16.0 software system (SPSS, Chicago, IL) was used for statistical analysis. Data are expressed as the mean ± standard error (S.D.). The differences between groups were analyzed using a Student t test when only 2 groups or 1-way analysis of variance when more than 2 groups were compared. Kaplan-Meier method and log-rank test were performed for patients' survival analyses. All experiments were run in triplicate. *P* < 0.05 is considered statistically significant.

## SUPPLEMENTARY FIGURES


