# Supplementary material for: A long noncoding RNA AB073614 promotes tumorigenesis and predicts poor prognosis in ovarian cancer
Source: Oncotarget. 2015 Jul 30;6(28):25381–9. doi: 10.18632/oncotarget.4541 (PMC4694838; doi:10.18632/oncotarget.4541)
Supplement: Supplementary file 1 [file oncotarget-06-25381-s001.pdf]

SUPPLEMENTAY FIGURES

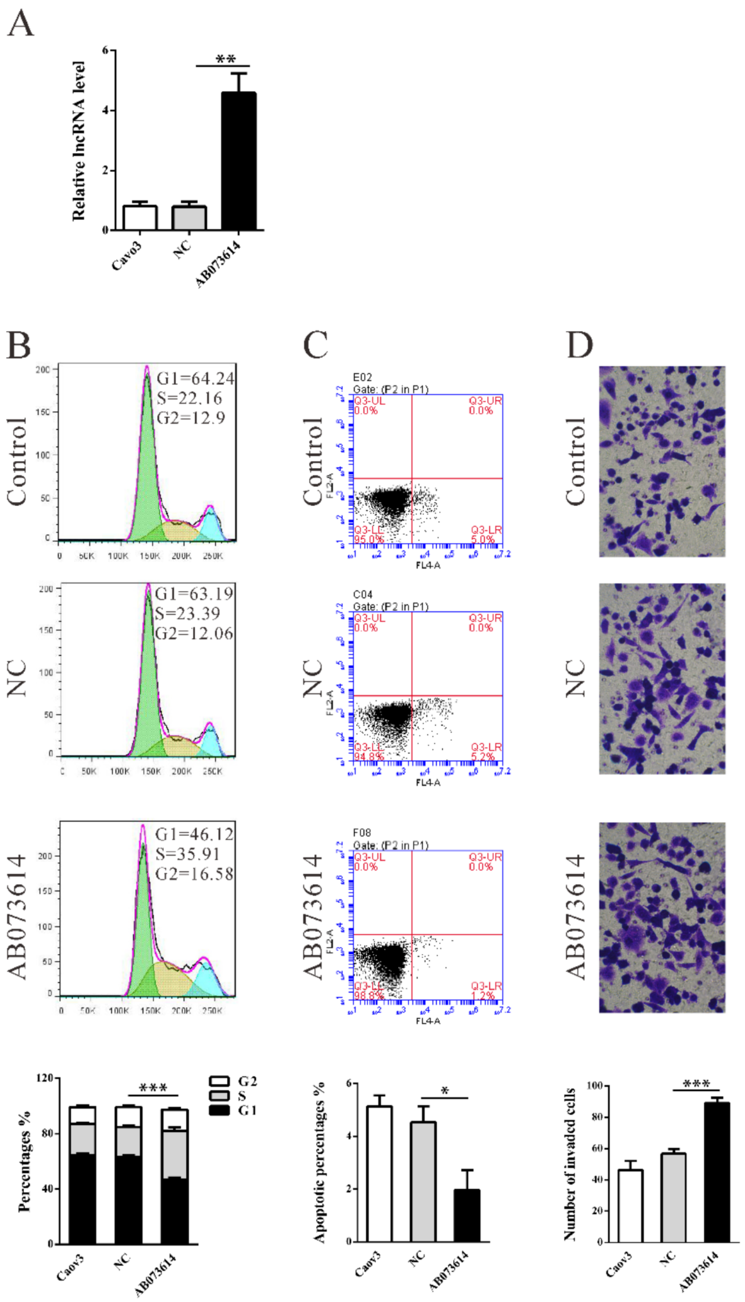

Supplementary Figure S1: Effects of LncRNA AB073614 overexpression on cell cycle, cell apoptosis and cell invasion. **A.** The full-length AB073614 were cloned into the expression vector pCDH-MSCV-MCS-EF1-GFP (System Biosciences, Mountain View, CA, USA) and transfected into Caov3 cells. AB073614 overexpression resulted in accelerated G1/S phase transition **B.** a notable decrease of apoptosis **C.** and a significant increase of cell invasion **D.**

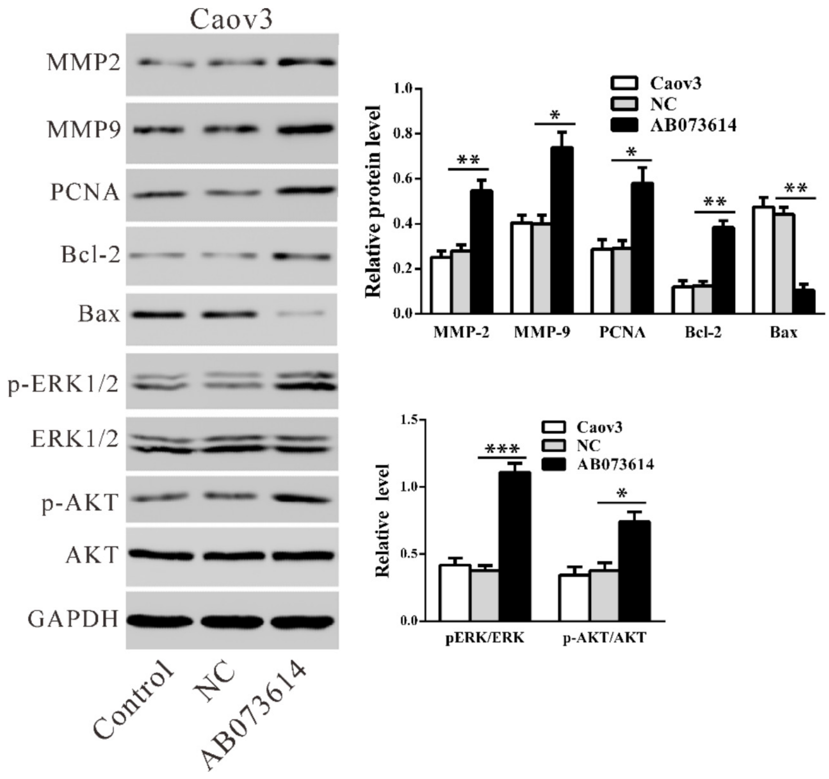

Supplementary Figure S2: Signal pathway and key moderators in tumor progression were determined by western blotting. Data were presented as the mean value from three independent experiments ± S.D. *$P < 0.05$, **$P < 0.01$, ***$P < 0.001$.
